# Listening through hearing aids affects spatial perception and speech intelligibility in normal-hearing listenersa)

**DOI:** 10.1121/1.5078582

**Published:** 2018-11-20

**Authors:** Jens Cubick, Jörg M. Buchholz, Virginia Best, Mathieu Lavandier, Torsten Dau

**Affiliations:** Hearing Systems Group, Department of Electrical Engineering, Technical University of Denmark, Ørsteds Plads, Building 352, 2800 Kongens Lyngby, Denmark; Department of Linguistics, Australian Hearing Hub, 16 University Avenue, Macquarie University, New South Wales 2109, Australia; Department of Speech, Language and Hearing Sciences, Boston University, Boston, Massachusetts 02215, USA; Univ Lyon, ENTPE, Laboratoire Génie Civil et Bâtiment, Rue M. Audin, F-69518 Vaulx-en-Velin, France; Hearing Systems Group, Department of Electrical Engineering, Technical University of Denmark, Ørsteds Plads, Building 352, 2800 Kongens Lyngby, Denmark

## Abstract

Cubick and Dau [(2016). Acta Acust. Acust. **102**, 547–557] showed that speech reception thresholds (SRTs) in noise, obtained with normal-hearing listeners, were significantly higher with hearing aids (HAs) than without. Some listeners reported a change in their spatial perception of the stimuli due to the HA processing, with auditory images often being broader and closer to the head or even internalized. The current study investigated whether worse speech intelligibility with HAs might be explained by distorted spatial perception and the resulting reduced ability to spatially segregate the target speech from the interferers. SRTs were measured in normal-hearing listeners with or without HAs in the presence of three interfering talkers or speech-shaped noises. Furthermore, listeners were asked to sketch their spatial perception of the acoustic scene. Consistent with the previous study, SRTs increased with HAs. Spatial release from masking was lower with HAs than without. The effects were similar for noise and speech maskers and appeared to be accounted for by changes to energetic masking. This interpretation was supported by results from a binaural speech intelligibility model. Even though the sketches indicated a change of spatial perception with HAs, no direct link between spatial perception and segregation of talkers could be shown.

## I. INTRODUCTION

In terms of speech intelligibility, hearing-aid (HA) users usually benefit most from their HAs in low-noise acoustic scenarios with a single talker. In more challenging acoustic situations, such as a social gathering in a crowded room, they typically have difficulties following a conversation (Bronkhorst, 2000), whereas normal-hearing listeners perform well almost effortlessly. Cherry (1953) introduced the term “cocktail party” to refer to such situations, where a listener attempts to understand a target speaker among various competing interferers. It has been demonstrated that spatial auditory cues are utilized by the auditory system to facilitate good intelligibility in these situations, such that interferers cause less masking when they are spatially separated from the target talker in terms of their azimuthal position (Hawley *et al.*, 2004; Plomp, 1976) or distance (Westermann and Buchholz, 2015). In the case of spatially-separated sources, speech intelligibility can be improved compared to collocated sources due to “better-ear” listening, where the sound at one ear, at a given moment, may provide an improved target-to-masker ratio, and/or due to the benefit of binaural unmasking, which improves the “internal” target-to-masker ratio (often conceptualized as an equalization-cancellation process; Durlach, 1972). Both strategies, better-ear listening and binaural unmasking, have been considered in various speech intelligibility modelling approaches (e.g., Beutelmann and Brand, 2006; Beutelmann *et al.*, 2010; Lavandier and Culling, 2010; Wan *et al.*, 2010; Rennies *et al.*, 2011; Lavandier *et al.*, 2012; Wan *et al.*, 2014; Chabot-Leclerc *et al.*, 2016) and are thought to reduce the effects of energetic masking (EM) of the target sound by the interferer(s).

However, some effects of typical cocktail-party scenarios on speech intelligibility cannot be accounted for in terms of EM. The term informational masking (IM) has been introduced to describe interference effects that reduce target intelligibility even in the case of sufficient target energy (for a review, see Kidd and Colburn, 2017). IM can refer to both difficulties in segregating speech mixtures (i.e., determining which parts belong to the target speech) and difficulties in terms of attending to a specific source in the sound mixture (i.e., overcoming confusion or distraction; Shinn-Cunningham, 2008). Spatial information regarding the target and the interferers in a speech mixture can strongly affect the amount of IM such that sound sources that are perceived as spatially separate objects are easier to segregate and attend to selectively (e.g., Freyman *et al.*, 1999). Spatial separation can be particularly effective when there is little other information available to separate the competing sounds (e.g., when the competing voices are of the same gender and/or have approximately the same sound pressure level). In fact, the magnitude of the “spatial release from IM” can even be larger than the “spatial release from EM” (e.g., Kidd *et al.*, 2005). Moreover, it appears that any cue that supports the perception of spatial separation of the target and the interferer(s) is sufficient to provide a release from IM. Such a release has been reported for interaural time differences (ITDs) and interaural level differences (ILDs) alone (e.g., Glyde *et al.*, 2013), monaural spectral cues associated with a separation in distance and elevation (e.g., Brungart and Simpson, 2002; Martin *et al.*, 2012; Westermann and Buchholz, 2015; Westermann and Buchholz, 2017a) and for illusory separation (e.g., Freyman *et al.*, 1999).

Because of the importance of spatial information for reducing EM and IM, any degradation of the spatial cues caused by a hearing loss and/or HA signal processing could potentially impair speech intelligibility in a cocktail-party like environment. A number of studies have explored the possibility that hearing loss impedes spatial perception, e.g., in terms of localization ability (Noble *et al.*, 1994; Lorenzi *et al.*, 1999; Best *et al.*, 2010; Best *et al.*, 2011; Hassager *et al.*, 2017) or ITD discrimination performance (e.g., Durlach *et al.*, 1981; Strelcyk and Dau, 2009; Spencer *et al.*, 2016). Furthermore, several studies have suggested that HAs can disrupt the auditory cues involved in spatial perception (Van den Bogaert *et al.*, 2006; Wiggins and Seeber, 2012; Akeroyd and Whitmer, 2016; Cubick and Dau, 2016; Hassager *et al.*, 2017). For example, Hassager *et al.* (2017) showed that localization accuracy in a moderately reverberant room was substantially degraded as a consequence of fast-acting dynamic-range compression in bilateral HAs, independent of whether the compression was synchronized across the two HAs or not. The distortions were attributed to the stronger amplification of the low-level portions of the (speech) signals that were dominated by early reflections and reverberation, relative to the higher-level direct sound components. As a result, increased diffuseness of the perceived sound and broader, sometimes internalized (“inside the head”) sound images, as well as sound image splits of a single speech source were observed both in normal-hearing and hearing-impaired listeners. However, the effects of these distortions on speech intelligibility were not investigated in that study. Cubick and Dau (2016) measured speech reception thresholds (SRTs) in normal-hearing listeners using omnidirectional regular production HAs with linear (i.e., level-independent) amplification. They found that amplification (relative to no amplification) increased SRTs by about 4 dB, i.e., degraded speech intelligibility, in a setting with spatially distributed sources inside a classroom. The study did not provide a fully conclusive explanation for the elevated SRTs. However, some of the listeners in that study reported a degraded spatial perception of the acoustic scene in the conditions with HA processing; the auditory images associated with the sound sources were often broader with HAs and sometimes perceived to be closer to the head than to the actual source position. These findings suggested that the elevated SRTs might, at least partly, reflect the reduced ability of the listeners to perceptually separate the target and interfering sounds due to disrupted localization cues.

Inspired by Cubick and Dau (2016), the current study investigated the potential effect of degraded spatial cues when listening through HAs on SRTs in spatially separated masking conditions. To do so, a very basic amplification scheme was used, that included linear gain and no sophisticated signal processing. This choice was made such that the only distortion of the incoming sound would be caused by the position of the microphones above the ears, which modifies the spatial cues compared to natural listening. This enabled a test of whether spatial distortions and elevated SRTs as in Cubick and Dau (2016) would be found even in the absence of effects related to specific signal processing schemes. Furthermore, it was investigated to what extent HAs affect the amount of IM (versus EM) in a complex acoustic setting with several interferers. SRTs were measured in a room with a target speaker in front of the listener and three interferers. The interferers were either competing talkers (potentially causing a large amount of IM) or noises (producing little if any IM), which were either spatially distributed around the listener (at +/− 90 and 180°) or collocated with the target source. In the extreme case, if the HAs were to completely remove all spatial information, no spatial release from masking (SRM) would be expected for either mixture. On the other hand, if the HAs were to distort the spatial information enough to disrupt the listeners' ability to perceptually separate the target and the interferer signals, then this might reduce the spatial release from IM and the impact would primarily be seen in the case of speech interferers. To characterize the influence of HAs on the spatial perception of the acoustic scenes in the horizontal plane, the same listeners were also asked to draw sketches to indicate the position and spatial distribution of the sound images they perceived using a method inspired by earlier studies (Plenge, 1972; Blauert and Lindemann, 1986; Cubick and Dau, 2016).

## II. METHODS

### A. Listeners

Ten native speakers of Australian English participated in the experiment. Most listeners were either students from Macquarie University or employed at the National Acoustic Laboratories. The average age of the listeners was 31 years. All listeners were required to have pure tone audiometric thresholds within 20 dB hearing level at audiometric frequencies between 125 Hz and 6 kHz. If a listener did not have a recent audiogram, an audiogram was measured before the experiment. All listeners received written information about the experiment and gave informed consent prior to testing. The experiments were approved by the Australian Hearing Human Research Ethics Committee. Listeners who were not employed at the National Acoustic Laboratories received a small gratuity in compensation for their travel expenses.

### B. Stimuli and apparatus

#### 1. Stimuli

For the target sentences in the SRT measurements, a speech corpus based on the Bamford–Kowal–Bench (BKB) sentence material (Bench *et al.*, 1979) was used. This open-set corpus consists of 1280 short, meaningful sentences with a simple syntactical structure, which are divided into 80 lists of 16 sentences each. The sentences are spoken by a female Australian-English talker. In the speech-on-speech conditions, recordings of three monologues were used as maskers (spoken by three female talkers different from the target). For speech-in-noise conditions, three instances of stationary speech-shaped noise (SSN) were generated with spectra that matched the individual long-term magnitude spectra of the three interfering talkers. To do so, a 2048-tap finite impulse response filter was derived from the difference between the spectrum of a white Gaussian noise sample and the estimated spectrum of the interfering talker. Convolving this difference filter with the white noise yielded the SSN.

#### 2. Hearing aids

The HAs used in the experiment were based on the premise that the highest possible sound quality achievable with common HA hardware should be provided, such that, ideally, the only influence on the ear signal compared to the unaided condition would be related to the provision of gain and the position of the microphones. A real-time HA processing platform was used that was developed in-house and that was run on a separate computer. The system used the front microphones and the receivers of standard behind-the-ear HA shells (Phonak Ambra). The microphone signals were amplified by a custom-made preamplifier and then fed into the computer via an RME Fireface UC audio interface. After the real-time processing, the output signal was sent to a calibrated limiter that interrupted the signal if it exceeded a sound pressure level of 85 dB. From here, the signal reached the HA receiver, which was coupled to the listeners' ears via tubes with foam plugs. The only HA processing used in the experiment was the application of a linear, frequency-independent (“flat”) gain on the omnidirectional microphone signal of the two front microphones of the HAs. The gain was adjusted in the software of the real-time platform to provide an approximately constant insertion gain of 10 dB across all frequencies between 63 Hz and 10 kHz, evaluated on a 2 cc coupler in a Siemens Unity 2 HA measurement box. The same gain settings were used for all participants. In all conditions with HAs, the playback level of the loudspeakers was reduced by 10 dB to keep the sound pressure level at the listener's ears approximately constant between conditions with and without HAs.

### C. Experimental procedure

#### 1. Speech intelligibility

The experiment was conducted in a sound-treated listening room with a reverberation time T30 of about 200 ms. The listeners were seated in the centre of a 1.3 -m radius ring of 16 Genelec 8020 loudspeakers. The stimuli were played from a computer running Matlab and delivered through an RME Fireface UFX audio interface and two RME ADI 8 DS 8-channel digital/analogue converters. During the experiment, only four of the 16 loudspeakers were used for playback. The target sentences were always presented from the front (0°), 1 s after a 200-ms long 1 kHz tone burst. The three maskers (speech or SSN) were presented continuously either from three loudspeakers at +/− 90° and 180° or from the same loudspeaker as the target sentences.

The target speech and the interferers were calibrated using an omnidirectional measurement microphone (Brüel & Kjær 4134) at the listening position. The masker level was kept constant at 65 dB (A) throughout the experiment, whereas the level of the target sentences was adapted using the 1-up-1-down procedure described in Keidser *et al.* (2013b). Each threshold was determined using 16–32 sentences. Each run lasted until either the standard error for the threshold estimate was below 0.8 dB or the maximum number of 32 sentences was reached. The experimenter was seated inside the test room, but outside the loudspeaker ring, and scored the correctly understood morphemes on a laptop that remote-controlled the PC used for stimulus generation.

#### 2. Spatial perception

Similar to the procedure in Cubick and Dau (2016), the listeners were asked in each run to draw the perceived position (both in angle and distance) and the extent of the target and masker sounds on a template depicting the listening setup with a schematic head in the middle indicating the listener's position and a circle indicating the radius of the loudspeaker ring. The listeners were given time to make the drawings in the beginning of each run, after the presentation of the first sentence. Some listeners updated their drawings during the run after hearing more samples of the stimuli.

### D. Stimulus conditions

Overall, eight conditions were tested. The three interferers were either speech or SSN, they were either spatially collocated with the target speech or separated, and the listeners either wore HAs (aided) or not (unaided). All listeners were tested twice in each of the resulting eight combinations. The experimental conditions were counterbalanced across subjects based on a Latin Square Design with the only restriction that the four aided and the four unaided conditions were always tested in consecutive runs. This was done to avoid listeners taking off and inserting the HAs more often than necessary, which could cause variability in HA positioning. The testing took part either in one session with a total duration of about two hours, or in two separate sessions of about 1 h 15 min each, depending on the listener's preference. Regular breaks were provided.

### E. Spatial cue analysis

To evaluate the acoustic effect of the BTE HAs on the spatial cues provided to the subjects, the ear signals as they occurred in the experiment were simulated using a Brüel & Kjær 4128 head-and-torso simulator (HATS). Binaural room impulse responses were measured at the listening position with and without HAs placed on HATS for all loudspeakers used in the experiment. The impulse responses were measured with two repetitions of a 6-s logarithmic sine sweep (Müller and Massarani, 2001) and truncated to a length of 300 ms for the analysis. To compensate for level differences between the left and the right ear of the HATS, the first 3.85 ms of the impulse responses of the left and right ear (corresponding to the direct sound from the front loudspeaker before the first room reflection) were filtered with the long-term magnitude spectrum of the target speech. The root mean square (RMS) values of the resulting filtered direct sound signals were compared and the signals were adjusted to have the same RMS. The resulting correction factor between left- and right-ear signals was subsequently applied to all recorded signals. The target sentences and interferer signals were convolved with the adjusted impulse responses and the resulting left and right ear signals for each source were used in the following analyses.

**FIG. 1.**
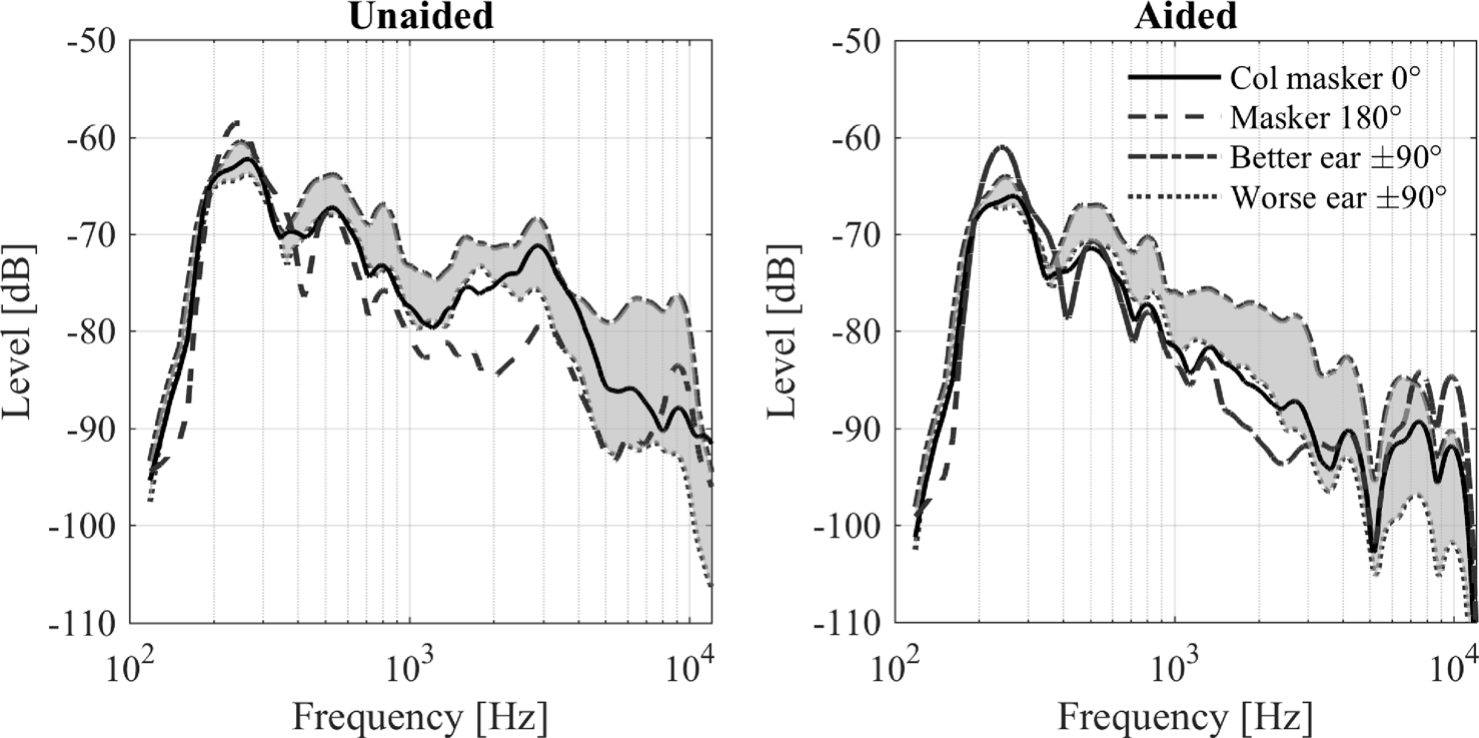
Long-term power spectra of the different masker signals at the ears of the HATS in the unaided (left panel) and aided (right panel) condition. The shaded area shows the averaged ILD for the two maskers at +/− 90°, the solid line indicates the average spectrum of the collocated maskers, and the dashed line shows the spectrum of the 180° masker averaged across ears.

For the spatial cue analysis, the long-term power spectra of the individual speech maskers at the two ears were calculated. The effect of the HAs on the long-term spectra is shown in Fig. 1, either averaged across left and right ear for the 0° (solid lines) and 180° (dashed lines) conditions or averaged across the ipsi- (dash-dotted lines) and contralateral ears (dotted lines) for the +90° and −90° conditions. The long-term spectra of the noise maskers are essentially identical to the ones of the speech maskers and are therefore not shown here. Also, the long-term spectrum of the target speech was similar to that of the 0° masker and is therefore not shown here. The BTE microphone placement mainly removed the ear canal resonance at around 2–3 kHz, which is seen in the unaided response (left panel), but absent in the aided response (right panel). It also generally decreased the energy towards higher frequencies. The ILD for the +90° and −90° masker, as indicated by the grey-shaded area, increased on average by 5 dB in the aided condition for frequencies above about 2 kHz. The ILD for the 0° and 180° masker was rather small for frequencies up to about 8 kHz and did not change significantly in the aided condition. The ear spectra were therefore averaged across ears.

The ITDs and the interaural coherence were computed using the Two!Ears auditory model (Two!Ears, 2017). The ITDs for the lateral maskers at the HA microphones were slightly reduced when compared to the in-ear microphones, but showed no other systematic difference. Also, the interaural coherence was not systematically affected by the HAs, except for a slight reduction at frequencies above 2–3 kHz.

### F. Modelling

In order to better understand the influence of the HAs on speech intelligibility in the present experiment, a model was used to quantify the amount of EM in the tested conditions. An updated implementation of the model proposed by Collin and Lavandier (2013) was used, which predicts binaural speech intelligibility in the presence of multiple non-stationary noises. It combines the effects of better-ear listening and binaural unmasking and is based on two inputs, the ear signals generated by the target, and the ear signals generated by the sum of all interferers. Based on these inputs, the model computes the better-ear target-to-interferer ratio and the binaural unmasking advantage in frequency bands, and finally produces the (broadband) effective target-to-interferer ratio in the corresponding condition (Jelfs *et al.*, 2011; Lavandier *et al.*, 2012), referred to as the “binaural ratio” in the following. Binaural ratios are inversely proportional to SRTs, such that high binaural ratios correspond to low SRTs. The predicted differences between conditions in terms of (inverted) binaural ratios were directly compared to corresponding SRT differences, without any fitting of the model to the data. The predictions in Collin and Lavandier (2013) were based on short-term predictions averaged across time, similar to Beutelmann *et al.* (2010) and Rhebergen and Versfeld (2005). To avoid target speech pauses mistakenly leading to a reduction in predicted intelligibility, the model needs to consider interfering energy as a function of time and target speech energy averaged across time (Collin and Lavandier; 2013). Instead of replacing the target speech by a stationary signal with a similar long-term spectrum and interaural parameters and applying the short-term analysis on this signal, as done by Collin and Lavandier, the present implementation of the model computes the long-term statistics of the target only once (see below) and combines these statistics with the short-term spectrum and interaural parameters of the noise to compute the better-ear and binaural unmasking components within each time frame (before averaging). The model used 24-ms half-overlapping Hann windows as time frames with an effective duration of 12 ms (Beutelmann *et al.*, 2010) and a gammatone filterbank (Patterson *et al.*, 1987) with two filters per equivalent rectangular bandwidth (Moore and Glasberg, 1983). A ceiling parameter corresponding to the maximum better-ear ratio allowed by frequency band and time frame was introduced to avoid the target-to-masker ratio tending to infinity in interferer pauses. This ceiling parameter was set to 20 dB here. Moreover, the binaural unmasking advantage was set to zero if the interferer power was zero at one of the ears in the considered band and frame.

The predictions presented here were computed from the ear signals described in Sec. II E, using two minutes of the masker signal in each of the eight tested conditions. The target was represented by averaging 144 target sentences, whereby the first 680 ms were omitted and all sentences were truncated to the duration of the shortest sentence. The RMS power of the averaged signal was then equalized to that of the corresponding collocated maskers.

## III. RESULTS

### A. Speech intelligibility

**FIG. 2.**
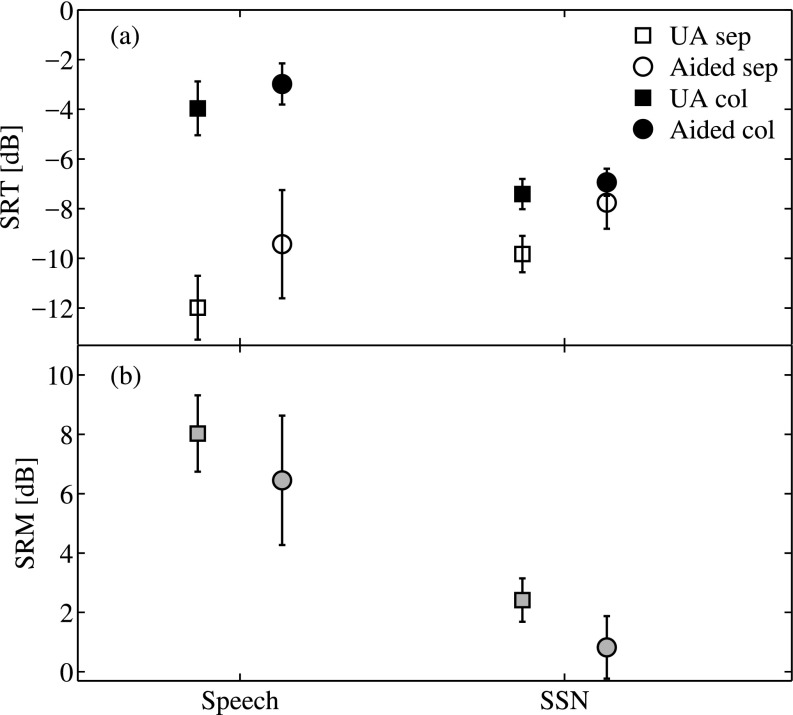
(a) Average SRTs and standard deviation for unaided (UA, squares) and aided (circles) conditions using speech interferers (left) or SSNs (right), for collocated (col, black filled symbols), and separated maskers (sep, open symbols). (b) Average spatial release from masking (grey filled symbols) and standard deviation across listeners for the two masker types and HA conditions.

Figure 2(a) shows the mean SRTs and standard deviation across participants for the unaided (squares) and the aided conditions (circles) for both the spatially separated (open symbols) and the collocated case (black filled symbols). The results for the speech interferers are shown on the left and the results obtained with SSN are shown on the right. The lowest SRT of −12 dB was observed in the unaided condition with separated speech interferers. With HAs, the threshold increased for this configuration by 2.5 dB. The average unaided threshold with separated SSN interferers was −9.8 dB, and thus 2.2 dB higher than with the speech interferers. With HAs, the SRT obtained with separated SSN increased by 2 dB to −7.8 dB (i.e., 1.7 dB above that obtained with speech interferers).

The thresholds for the collocated conditions were in all cases higher than for the corresponding condition with separated maskers. The SRM, shown in Fig. 2(b), was calculated as the difference between the individual separated and collocated SRTs. The highest average SRM (8 dB unaided, 6.5 dB aided) was found in the conditions with the speech interferers (left). In the case of SSN (right), the SRM was much lower (2.4 dB unaided, 0.8 dB aided).

A linear mixed effects model was fitted to the SRT data with the three factors “Masker type,” “Spatial distribution,” and “HA condition.” The full model with all interaction terms was then simplified by removing the non-significant three-factor interaction. The subsequent analysis of variance (ANOVA) showed that all three main effects HA condition [F(1,144)= 138.84, p < 0.0001], Masker type [F(1,144) = 7.7513, p= 0.0061], and Spatial distribution [F(1,144) = 522.74, p < 0.0001], and the two-factor interactions between HA condition and Spatial distribution [F(1,144) = 15.27, p = 0.0001] and Masker type and Spatial distribution [F(1,144) = 191.92, p < 0.0001] were significant. Only the interaction between HA condition and Masker type was not significant [F(1,144)= 1.51, p = 0.2213].

Similarly, a linear mixed-effects model was fitted to the SRM data with factors HA condition and Masker type. Here, the ANOVA showed significant main effects for both HA condition [F(1, 67) = 9.63, p = 0.0028] and Masker type [F(1, 67) = 122.00, p < 0.0001], but no significant interaction [F(1, 67) = 0.0006, p = 0.9804]. This indicates that the SRM in the SSN conditions was significantly lower than in the speech conditions, and HAs reduced the amount of SRM similarly in the SSN and the speech conditions.

### B. Spatial perception

**FIG. 3.**
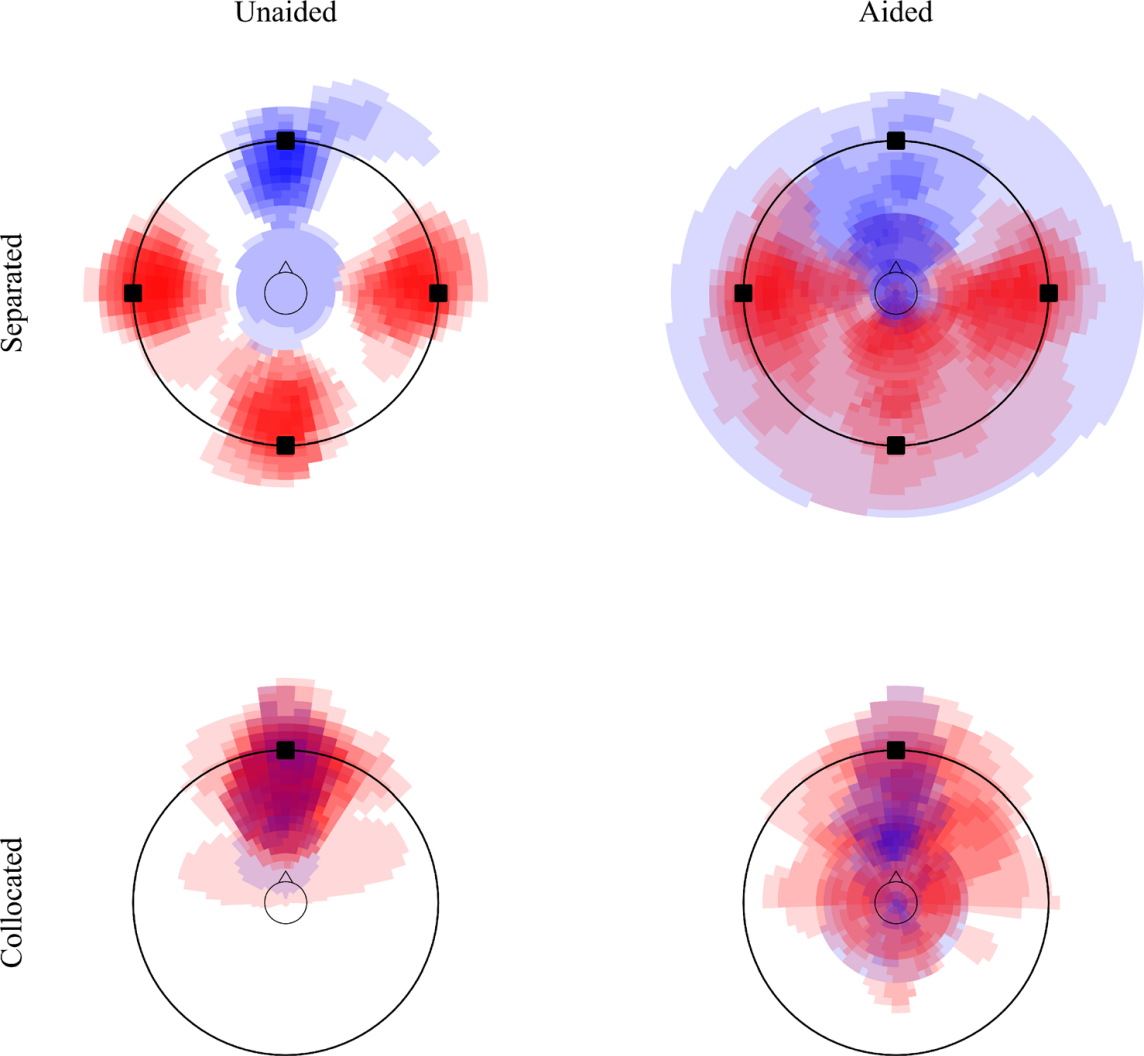
(Color online) Superimposed images of the perceived positions of the sound sources for target speech (blue) and interfering talkers (red) for the unaided conditions (left column) and aided conditions (right column), and the separated conditions (top row) and collocated conditions (bottom row). The two circles indicate the listener's head (inner) and the loudspeaker ring (outer) as shown in the sketch template provided to the listeners during the experiment. The black squares indicate the positions of the loudspeakers through which the stimuli were presented.

Figure 3 shows the provided template and the digitized data from the position sketches collected from all listeners for the four conditions with speech interferers. The outer circle indicates the ring of loudspeakers with squares indicating the loudspeakers that were actually playing in the corresponding condition. The inner circle represents the listener's head. Pixels representing the target sound are shown in blue, pixels belonging to the interferers are shown in red (colour online). All images were superimposed; therefore, areas of higher saturation represent areas that were marked as belonging to the auditory image by more listeners.

In the unaided separated case (top left panel), all listeners drew clearly separated images for the target and the three distracting talkers. Only one listener sketched the target sound image as being close to and inside the head in both repetitions of this condition. Compared to the unaided condition, the corresponding sketches for the aided separated condition (top right panel) indicate a much larger variability in the data. In many cases, not only was the image position more variable across listeners, but the images were also often broader and differed in their perceived distance. Several listeners indicated that they had perceived the target and/or the interferers inside their head, or to be spread indistinguishably in the whole room. In the collocated conditions (bottom panels), most listeners indicated the target and the interferer sound images to be somewhere between their head and the front loudspeaker in the unaided condition. Again, with HAs, the data showed more variability where, e.g., the interfering sounds were perceived from different directions and resulted in broader auditory images and sometimes internalized percepts.

**FIG. 4.**
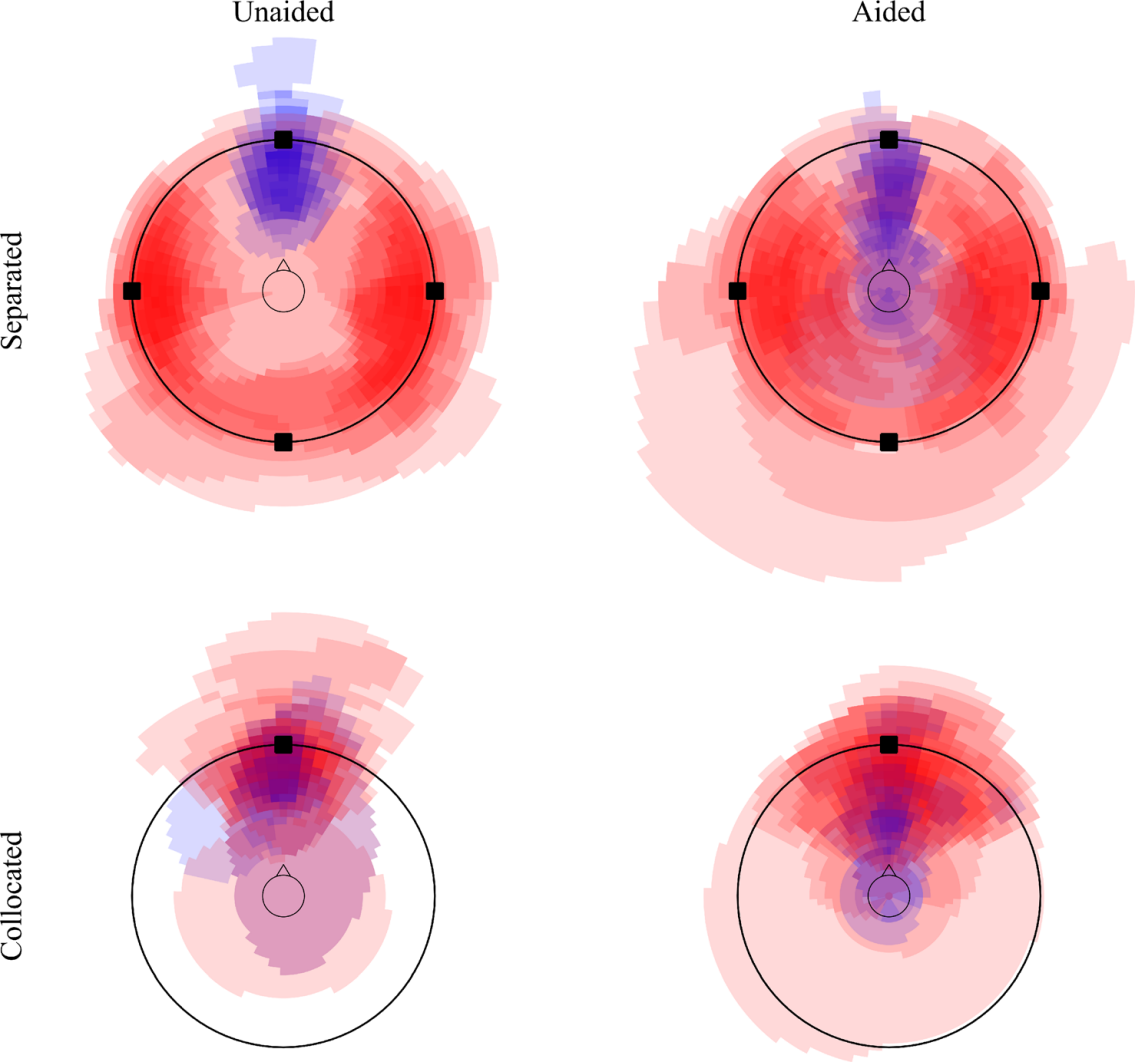
(Color online) Superimposed images of the perceived positions of the sound sources for target speech (blue) and SSN (red) in the unaided conditions (left column) and the aided conditions (right column), and in the separated conditions (top row) and collocated conditions (bottom row).

Figure 4 shows the corresponding sketches for the conditions with SSN. One effect that cannot be seen from the figure is that, unlike in the conditions with speech interferers, all listeners indicated only one or two interfering sources in all SSN conditions. Apparently, the spectral differences between the noise maskers were not sufficient to perceive them as separate auditory objects, and the three noise maskers were fused into one or two objects instead. In the unaided separated condition (top left panel), the target speech again yielded sharply focussed and fairly narrow auditory images between the listener and the loudspeaker at 0°, as seen by the narrow blue “wedge.” All listeners perceived the target externalized in this condition. In the individual sketches (not shown), the three noise sources were fused into either two wide auditory images to the left and right of the listener, or into a single auditory image behind the listener or perceived all around the room. In the aided separated condition (top right panel), the sound sources often changed their position compared to the unaided condition. Some listeners perceived the target inside their heads or behind them. Also, the position of the noise sound images often moved. In some cases, the images were indicated closer to the listener or all over the room.

In the unaided collocated condition (bottom left panel), the sketches show a larger spread than in the corresponding condition with interfering talkers (Fig. 3, bottom left panel), but in the majority of cases, the auditory images of both target and masker were perceived in the front. In the aided collocated condition (bottom right panel), there was a tendency for the noise maskers to create a larger auditory image than the target speech, and for the noise sources to be perceived far away and broad, whereas the auditory image of the target speech tended to be closer to the listener and more compact. Interestingly, there were some cases where the target and maskers were perceived as more separated in the aided than in the unaided condition.

### C. Modelling

**FIG. 5.**
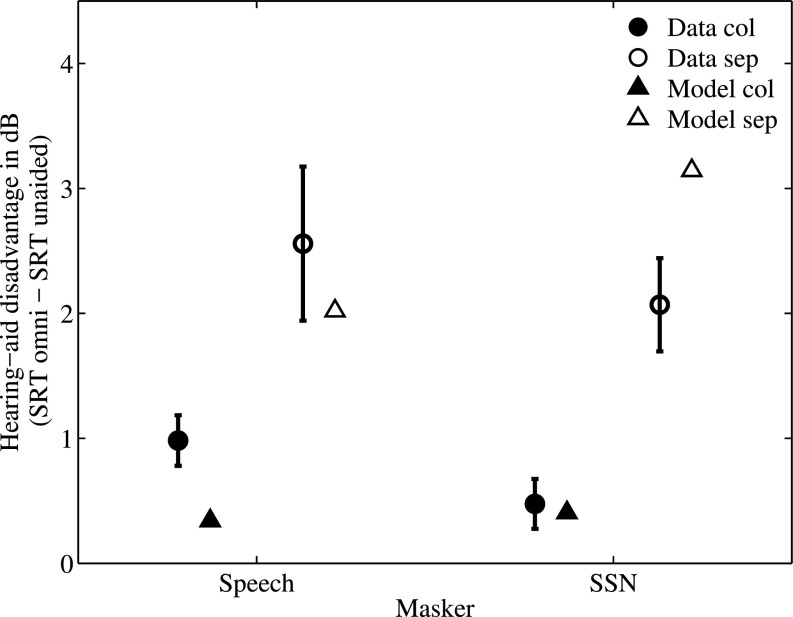
Hearing-aid disadvantage in dB evaluated as the difference between the SRTs in the aided and unaided conditions for the SSN and speech interferers in the collocated and separated conditions. The circles represent the measured average disadvantage across listeners; the error bars indicate the standard errors. The triangles indicate the disadvantage predicted by the model.

The speech intelligibility results showed that using omnidirectional HAs led to an increase in SRT in all tested conditions. Figure 5 presents this “HA disadvantage” calculated for each condition (collocated and spatially separated, speech and SSN interferers) as the difference between the SRTs in the aided and unaided conditions. The triangles indicate the predicted values of the HA disadvantage obtained from the model. The average and maximum prediction errors (absolute difference between measured and predicted disadvantages) across conditions were 0.6 and 1.1 dB, respectively. The deleterious effect of the HAs in the tested conditions is predicted reasonably well by the model, indicating that this effect is most likely associated with EM, since the model does not account for IM.

## IV. DISCUSSION

The lowest SRTs in this study were found in the unaided condition with spatially separated speech interferers. In the corresponding condition with SSN interferers, SRTs were higher by 2.2 dB on average. This difference can be attributed to “listening in the dips” in the case of the speech interferers that exhibit fluctuating envelopes (e.g., Festen and Plomp, 1990) whereas the SSN maskers offer fewer opportunities for dip listening and hence represent a more effective masker in the separated condition.

For the collocated interferers, the HA processing did not have a detrimental effect on speech intelligibility. In contrast, for the separated interferers, speech intelligibility was generally worse with HAs than without HAs, independent of the type of interferer, consistent with the findings of Cubick and Dau (2016). This indicates that it might indeed be the distortions to the spatial cues caused by the HAs that impede the segregation of the target and interferers, since the detrimental effect of HAs was predominantly seen in those conditions in which spatial cues are crucial. However, the average SRT increase in this experiment was only 2.5 dB for the speech interferers and 2 dB for the SSN interferer, compared to the 4 dB (with SSN) reported in Cubick and Dau (2016). This difference might be due to the fact that, in the present study, a PC-based real-time processing platform was used, allowing for a wider bandwidth (here 12 kHz), a lower noise floor, and better overall sound quality than the regular production HAs used in Cubick and Dau (2016). Better sound quality might have improved aided speech perception. Another difference between the two studies is that the room considered in Cubick and Dau (2016) was more reverberant than the one in the present study (0.5 s compared to 0.2 s). This increased amount of reflected energy in Cubick and Dau (2016) might have been particularly detrimental in the aided listening conditions since the natural directivity of the pinna is lost when listening with behind-the-ear HAs, which would otherwise attenuate sounds from the back to some extent and thus emphasize the direct sound. Another difference between the two studies is that the loudspeakers in Cubick and Dau (2016) were placed at +/−112.5°, not at +/−90° as in the current study. However, since omnidirectional HA microphones located on the side of the head have a slightly higher sensitivity for lateral angles, interferers at +/−90° would be emphasized more than interferers at +/−112.5°, which should have led to an even *more* detrimental effect of HAs in the present study, contrary to what was observed.

The average SRTs in the collocated conditions were consistently higher than those in the separated conditions. In the unaided condition, the resulting SRM was larger for speech interferers (8 dB) than for SSN (2.4 dB). This is entirely consistent with previous studies (e.g., Kidd *et al.*, 2005; Marrone *et al.*, 2008; Westermann and Buchholz, 2015) and indicates that the speech interferers caused some IM in addition to EM that was released via spatial separation. In the aided condition, the SRM was reduced by 1.6 dB, irrespective of the type of interferer. This suggests that while release from IM was observed in this study, it did not change with the use of HAs. The reduction of SRM by the HAs can therefore be fully attributed to changes in EM, not IM.

In the model, the better-ear and binaural unmasking components are computed independently; hence their relative contribution to SRM can be evaluated. The predicted binaural unmasking advantage was neither influenced by the HA, nor by the type of interferer. It accounted for about 1.5 dB of the overall SRM. The predicted better-ear advantage was very small for the unaided SSN condition, indicating that there is no long-term better ear effect with a masker on either side of the listener, and very little opportunity for glimpsing within three unmodulated SSNs. The predicted better-ear advantage was −2 dB in the aided SSN condition. This might be explained by the fact that, at high frequencies, the listener's head acts as a small baffle for the omnidirectional HA microphones, thereby effectively amplifying the high frequencies for sounds coming from the sides, such that the effective SNR at the ears is worse in the case of spatially separated maskers than when the maskers are collocated with the frontal target speech. A long-term version of the model, which considers the whole duration of the signals instead of short-term predictions, provided very similar better-ear predictions, indicating that the better-ear disadvantage for SSNs is a long-term SNR effect rather than associated with short-term glimpsing.

The predicted better-ear benefit was larger for speech maskers than for SSN, both in the unaided condition (1.3 dB) and in the aided condition (2.3 dB). This is because the model is sensitive to advantageous SNR glimpses in one ear or the other with the fluctuating speech maskers. In addition to this “better-ear glimpsing” benefit, the model predicts the SRTs to be about 6 dB lower for collocated speech compared to collocated SSN, whereas the stationary model predicts similar SRTs, suggesting that “monaural glimpsing” was quite strong even with three speech maskers involved.

The model was also used to predict the spatial release from IM in the unaided and aided conditions involving the speech maskers. Since the model can only predict the effect of EM, not IM, this IM release was estimated as the difference between the measured and predicted SRMs for the speech maskers. It should be noted that EM prediction errors were thus incorporated in this IM release estimation. The predicted spatial release from IM was 5.1 dB in the unaided condition and 5.2 dB in the aided condition, supporting the hypothesis that spatial release from IM was probably not affected by the HA processing in the present study. This result was somewhat surprising, because the spatial release from IM has commonly been linked to the perceived spatial separation of the target and interferer signals (e.g., Freyman *et al.*, 1999), and the sketches in Fig. 3 suggest that this perceived spatial separation is reduced here for speech interferers when HAs are applied. It is possible that this reduction of the perceived spatial separation of the HA-processed stimuli was not substantial enough to affect speech intelligibility performance. The large spatial separation between the target and interferers may require major distortions of the spatial cues before any significant change in SRM can be observed. Marrone *et al.* (2008) and Jakien *et al.* (2017) indeed showed that even a change in spatial separation from 90° to 45° azimuth only has a minor effect on SRM.

It is important to note that this study focused on a very specific set of stimuli and conditions. Clearly, further studies would be useful for expanding the conclusions that can be made about the effect of HAs on spatial perception and speech intelligibility. For example, only normal-hearing listeners with no HA experience were tested in the present study. This lack of experience might account for some of the reduced performance in the aided conditions. To test for potential training effects, paired t-tests were applied to the data from the first and the second test run in each condition. A significant difference was only found for the aided condition with spatially separated speech maskers (1.9 dB, p = 0.0162), which resulted in a reduction of the detrimental effect of the HAs (cf. Fig. 5) by about 1–1.6 dB. The model prediction of 2 dB is thus closer to the measured detrimental effect after training than to the untrained or averaged data. Since speech intelligibility commonly exhibits high variability across lists (e.g., Keidser *et al.*, 2013a), additional data would be needed to reliably estimate the training effect and determine the long-term effect of the microphone placement above the ear.

The present study investigated whether the fundamental limitations of HAs might reduce the potential benefit a listener can get from using the devices. Testing normal-hearing listeners allowed the separation of these HA-related effects from the hearing-loss related challenges that hearing-impaired listeners experience, but ultimately, it is of course important to determine whether similar effects would also be observed in hearing-impaired listeners, particularly in listeners with HA experience. It is difficult to predict how the distortions of spatial cues that HAs cause (cf. Sec. II E) might interact with the impaired auditory system, which typically shows a reduced spectral and temporal resolution. Moreover, hearing-impaired listeners are known to benefit less from the spatial separation of competing sounds (e.g., Bronkhorst and Plomp, 1992; Marrone *et al.*, 2008) and thus have less to lose than listeners with normal hearing. It would also be interesting to conduct a similar experiment but with real HAs instead of the simplified HAs used in this study. Modern HAs with their highly non-linear and adaptive processing have been shown to affect binaural cues and spatial perception (e.g., Keidser *et al.*, 2006; Van den Bogaert *et al.*, 2006; Van den Bogaert *et al.*, 2008; Brown *et al.*, 2016). It can be expected that spatial perception would be even more distorted with such devices, which might have major consequences for the ability of listeners to segregate competing talkers. In such studies, it would be necessary to disentangle these potentially detrimental effects from the expected positive effects of processing schemes, such as noise reduction or beamforming algorithms.

While the distortions considered in the present study were accompanied by small energetic changes, they did not eliminate the ability of listeners to perceptually segregate the target and interfering sounds based on spatial location. Considering that substantial SRM has been observed for much smaller spatial separations as little as 15° (Marrone *et al.*, 2008) and that some SRM has been found even for 2° separation (Srinivasan *et al.*, 2016) in normal-hearing listeners, this may be because the sources were widely separated and the broader images were still sufficiently distinct from one another to support segregation. It would be interesting to investigate the effect of HA processing on speech intelligibility in conditions with interferers located more closely to the target speaker, where segregation is more challenging (e.g., Westermann and Buchholz, 2017b). Furthermore, future studies should consider testing speech intelligibility at higher SNRs that are more typical for real-life scenarios (Smeds *et al.*, 2015).

## V. SUMMARY AND CONCLUSION

In this study, it was found that listening through HAs led to distorted spatial perception and poorer speech intelligibility in normal-hearing listeners in conditions with spatially separated target and interfering sources. HAs reduced SRM equally for speech and noise maskers, suggesting that their detrimental effect can largely be explained by changes in EM, and that the spatial distortions were not sufficient to impede spatial release from IM. This finding was supported by binaural speech intelligibility modelling.

## References

[1] Akeroyd, M. A. , and Whitmer, W. M. (2016). “ Spatial hearing and hearing aids,” in *Hearing Aids*, edited by Popelka G. R., Moore B. C. J., Fay R. R., and Popper A. N. ( Springer International, Berlin), pp. 181–215.

[2] Bench, J. , Kowal, Å. , and Bamford, J. (1979). “ The BKB (Bamford-Kowal-Bench) sentence lists for partially-hearing children,” Brit. J. Audiol. 13(3), 108–112. 10.3109/03005367909078884 486816

[3] Best, V. , Carlile, S. , Kopčo, N. , and van Schaik, A. (2011). “ Localization in speech mixtures by listeners with hearing loss,” J. Acoust. Soc. Am. 129(5), EL210–EL215. 10.1121/1.3571534 21568377

[4] Best, V. , Kalluri, S. , McLachlan, S. , Valentine, S. , Edwards, B. , and Carlile, S. (2010). “ A comparison of CIC and BTE hearing aids for three-dimensional localization of speech,” Int. J. Audiol. 49(10), 723–732. 10.3109/14992027.2010.484827 20515424

[5] Beutelmann, R. , and Brand, T. (2006). “ Prediction of speech intelligibility in spatial noise and reverberation for normal-hearing and hearing-impaired listeners,” J. Acoust. Soc. Am. 120(1), 331–342. 10.1121/1.2202888 16875230

[6] Beutelmann, R. , Brand, T. , and Kollmeier, B. (2010). “ Revision, extension, and evaluation of a binaural speech intelligibility model,” J. Acoust. Soc. Am. 127(4), 2479–2497. 10.1121/1.3295575 20370031

[7] Blauert, J. , and Lindemann, W. (1986). “ Spatial mapping of intracranial auditory events for various degrees of interaural coherence,” J. Acoust. Soc. Am. 79(3), 806–813. 10.1121/1.393471 3958323

[8] Bronkhorst, A. W. (2000). “ The cocktail party phenomenon: A review of research on speech intelligibility in multiple-talker conditions,” Acta Acust. Acust. 86(1), 117–128.

[9] Bronkhorst, A. W. , and Plomp, R. (1992). “ Effect of multiple speechlike maskers on binaural speech recognition in normal and impaired hearing,” J. Acoust. Soc. Am. 92(6), 3132–3139. 10.1121/1.404209 1474228

[10] Brown, A. D. , Rodriguez, F. A. , Portnuff, C. D. F. , Goupell, M. J. , and Tollin, D. J. (2016). “ Time-varying distortions of binaural information by bilateral hearing aids: Effects of nonlinear frequency compression,” Trends Hear. 20, 1–15. 10.1177/2331216516668303 PMC5051674 27698258

[11] Brungart, D. S. , and Simpson, B. D. (2002). “ Within-ear and across-ear interference in a cocktail-party listening task,” J. Acoust. Soc. Am. 112(6), 2985–2995. 10.1121/1.1512703 12509020

[12] Chabot-Leclerc, A. , MacDonald, E. N. , and Dau, T. (2016). “ Predicting binaural speech intelligibility using the signal-to-noise ratio in the envelope power spectrum domain,” J. Acoust. Soc. Am. 140(1), 192–205. 10.1121/1.4954254 27475146

[13] Cherry, E. C. (1953). “ Some experiments on the recognition of speech, with one and with two ears,” J. Acoust. Soc. Am. 25(5), 975–979. 10.1121/1.1907229

[14] Collin, B. , and Lavandier, M. (2013). “ Binaural speech intelligibility in rooms with variations in spatial location of sources and modulation depth of noise interferers,” J. Acoust. Soc. Am. 134(2), 1146–1159. 10.1121/1.4812248 23927114

[15] Cubick, J. , and Dau, T. (2016). “ Validation of a virtual sound environment system for testing hearing aids,” Acta Acust. united Acust. 102(3), 547–557. 10.3813/AAA.918972

[16] Durlach, N. I. (1972). “ Binaural signal detection—Equalization and cancellation theory,” in *Foundations of Modern Auditory Theory* ( Academic, New York), Vol. 2, pp. 371–462.

[17] Durlach, N. I. , Thompson, C. L. , and Colburn, H. S. (1981). “ Binaural interaction in impaired listeners: A review of past research,” Audiology 20(3), 181–211. 10.3109/00206098109072694 7011289

[18] Festen, J. M. , and Plomp, R. (1990). “ Effects of fluctuating noise and interfering speech on the speech-reception threshold for impaired and normal hearing,” J. Acoust. Soc. Am. 88(4), 1725–1736. 10.1121/1.400247 2262629

[19] Freyman, R. L. , Helfer, K. S. , McCall, D. D. , and Clifton, R. K. (1999). “ The role of perceived spatial separation in the unmasking of speech,” J. Acoust. Soc. Am. 106(6), 3578–3588. 10.1121/1.428211 10615698

[20] Glyde, H. , Buchholz, J. M. , Dillon, H. , Cameron, S. , and Hickson, L. (2013). “ The importance of interaural time differences and level differences in spatial release from masking,” J. Acoust. Soc. Am. 134(2), EL147–EL152. 10.1121/1.4812441 23927217

[21] Hassager, H. G. , Wiinberg, A. , and Dau, T. (2017). “ Effects of hearing-aid dynamic range compression on spatial perception in a reverberant environment,” J. Acoust. Soc. Am. 141(4), 2556–2568. 10.1121/1.4979783 28464692

[22] Hawley, M. L. , Litovsky, R. Y. , and Culling, J. F. (2004). “ The benefit of binaural hearing in a cocktail party: Effect of location and type of interferer,” J. Acoust. Soc. Am. 115(2), 833–843. 10.1121/1.1639908 15000195

[23] Jakien, K. M. , Kampel, S. D. , Gordon, S. Y. , and Gallun, F. J. (2017). “ The benefits of increased sensation level and bandwidth for spatial release from masking,” Ear Hear. 38(1), e13–e21. 10.1097/AUD.0000000000000352 27556520 PMC5161636

[24] Jelfs, S. , Culling, J. F. , and Lavandier, M. (2011). “ Revision and validation of a binaural model for speech intelligibility in noise,” Hear. Res. 275(1), 96–104. 10.1016/j.heares.2010.12.005 21156201

[25] Keidser, G. , Dillon, H. , Convery, E. , and Mejia, J. (2013a). “ Factors influencing individual variation in perceptual directional microphone benefit,” J. Am. Acad. Audiol. 24, 955–968. 10.3766/jaaa.24.10.7 24384081

[26] Keidser, G. , Dillon, H. , Mejia, J. , and Nguyen, C.-V. (2013b). “ An algorithm that administers adaptive speech-in-noise testing to a specified reliability at selectable points on the psychometric function,” Int. J. Audiol. 52(11), 795–800. 10.3109/14992027.2013.817688 23957444

[27] Keidser, G. , Rohrseitz, K. , Dillon, H. , Hamacher, V. , Carter, L. , Rass, U. , and Convery, E. (2006). “ The effect of multi-channel wide dynamic range compression, noise reduction, and the directional microphone on horizontal localization performance in hearing aid wearers,” Int. J. Audiol. 45(10), 563–579. 10.1080/14992020600920804 17062498

[28] Kidd, G. , Mason, C. R. , Brughera, A. , and Hartmann, W. M. (2005). “ The role of reverberation in release from masking due to spatial separation of sources for speech identification,” Acta Acust. Acust. 91(3), 526–536.

[29] Kidd, G., Jr. , and Colburn, H. S. (2017). “ Informational masking in speech recognition,” in *The Auditory System at the Cocktail Party* ( Springer, Berlin), pp. 75–109.

[30] Lavandier, M. , and Culling, J. F. (2010). “ Prediction of binaural speech intelligibility against noise in rooms,” J. Acoust. Soc. Am. 127(1), 387–399. 10.1121/1.3268612 20058985

[31] Lavandier, M. , Jelfs, S. , Culling, J. F. , Watkins, A. J. , Raimond, A. P. , and Makin, S. J. (2012). “ Binaural prediction of speech intelligibility in reverberant rooms with multiple noise sources,” J. Acoust. Soc. Am. 131(1), 218–231. 10.1121/1.3662075 22280586

[32] Lorenzi, C. , Gatehouse, S. , and Lever, C. (1999). “ Sound localization in noise in hearing-impaired listeners,” J. Acoust. Soc. Am. 105(6), 3454–3463. 10.1121/1.424672 10380669

[33] Marrone, N. , Mason, C. R. , and Kidd, G., Jr. (2008). “ The effects of hearing loss and age on the benefit of spatial separation between multiple talkers in reverberant rooms,” J. Acoust. Soc. Am. 124(5), 3064–3075. 10.1121/1.2980441 19045792 PMC2736722

[34] Martin, R. L. , McAnally, K. I. , Bolia, R. S. , Eberle, G. , and Brungart, D. S. (2012). “ Spatial release from speech-on-speech masking in the median sagittal plane,” J. Acoust. Soc. Am. 131(1), 378–385. 10.1121/1.3669994 22280599

[35] Moore, B. C. J. , and Glasberg, B. R. (1983). “ Suggested formulae for calculating auditory-filter bandwidths and excitation patterns,” J. Acoust. Soc. Am. 74(3), 750–753. 10.1121/1.389861 6630731

[36] Müller, S. , and Massarani, P. (2001). “ Transfer-function measurement with sweeps,” J. Audio Eng. Soc. 49(6), 443–471.

[37] Noble, W. , Byrne, D. , and Lepage, B. (1994). “ Effects on sound localization of configuration and type of hearing impairment,” J. Acoust. Soc. Am. 95(2), 992–1005. 10.1121/1.408404 8132913

[38] Patterson, R. D. , Nimmo-Smith, I. , Holdsworth, J. , and Rice, P. (1987). “ An efficient auditory filterbank based on the gammatone function,” presented to the *Institute of Acoustics Speech Group on Auditory Modelling at the Royal Signal Research Establishment*, Vol. 2.

[39] Plenge, G. (1972). “ Über das Problem der Im-Kopf-Lokalisation” (“On the problem of in head localization),” Acustica 26(5), 241–252.

[40] Plomp, R. (1976). “ Binaural and monaural speech intelligibility of connected discourse in reverberation as a function of azimuth of a single competing sound source (speech or noise),” Acustica 34(4), 200–211.

[41] Rennies, J. , Brand, T. , and Kollmeier, B. (2011). “ Prediction of the influence of reverberation on binaural speech intelligibility in noise and in quiet,” J. Acoust. Soc. Am. 130(5), 2999–3012. 10.1121/1.3641368 22087928

[42] Rhebergen, K. S. , and Versfeld, N. J. (2005). “ A speech intelligibility index-based approach to predict the speech reception threshold for sentences in fluctuating noise for normal-hearing listeners,” J. Acoust. Soc. Am. 117(4), 2181–2192. 10.1121/1.1861713 15898659

[43] Shinn-Cunningham, B. G. (2008). “ Object-based auditory and visual attention,” Trends Cogn. Sci. 12(5), 182–186. 10.1016/j.tics.2008.02.003 18396091 PMC2699558

[44] Smeds, K. , Wolters, F. , and Rung, M. (2015). “ Estimation of signal-to-noise ratios in realistic sound scenarios,” J. Am. Acad. Audiol. 26(2), 183–196. 10.3766/jaaa.26.2.7 25690777

[45] Spencer, N. J. , Hawley, M. L. , and Colburn, H. S. (2016). “ Relating interaural difference sensitivities for several parameters measured in normal-hearing and hearing-impaired listeners,” J. Acoust. Soc. Am. 140(3), 1783–1799. 10.1121/1.4962444 27914394 PMC5035301

[46] Srinivasan, N. K. , Jakien, K. M. , and Gallun, F. J. (2016). “ Release from masking for small spatial separations: Effects of age and hearing loss,” J. Acoust. Soc. Am. 140, EL73–EL78. 10.1121/1.4954386 27475216 PMC5392088

[47] Strelcyk, O. , and Dau, T. (2009). “ Relations between frequency selectivity, temporal fine-structure processing, and speech reception in impaired hearing,” J. Acoust. Soc. Am. 125(5), 3328–3345. 10.1121/1.3097469 19425674

[48] Two!Ears (2017). “ Two!Ears auditory model 1.4,” 10.5281/zenodo.238761 (Last viewed November 7, 2018).

[49] Van den Bogaert, T. , Doclo, S. , Wouters, J. , and Moonen, M. (2008). “ The effect of multimicrophone noise reduction systems on sound source localization by users of binaural hearing aids,” J. Acoust. Soc. Am. 124(1), 484–497. 10.1121/1.2931962 18646992

[50] Van den Bogaert, T. , Klasen, T. J. , Moonen, M. , Van Deun, L. , and Wouters, J. (2006). “ Horizontal localization with bilateral hearing aids: Without is better than with,” J. Acoust. Soc. Am. 119(1), 515–526. 10.1121/1.2139653 16454305

[51] Wan, R. , Durlach, N. I. , and Colburn, H. S. (2010). “ Application of an extended equalization-cancellation model to speech intelligibility with spatially distributed maskers,” J. Acoust. Soc. Am. 128(6), 3678–3690. 10.1121/1.3502458 21218900 PMC3037771

[52] Wan, R. , Durlach, N. I. , and Colburn, H. S. (2014). “ Application of a short-time version of the equalization-cancellation model to speech intelligibility experiments with speech maskers,” J. Acoust. Soc. Am. 136(2), 768–776. 10.1121/1.4884767 25096111 PMC4144180

[53] Westermann, A. , and Buchholz, J. M. (2015). “ The effect of spatial separation in distance on the intelligibility of speech in rooms,” J. Acoust. Soc. Am. 137(2), 757–767. 10.1121/1.4906581 25698010

[54] Westermann, A. , and Buchholz, J. M. (2017a). “ The effect of hearing loss on source-distance dependent speech intelligibility in rooms,” J. Acoust. Soc. Am. 141(2), EL140–EL145. 10.1121/1.4976191 28253708

[55] Westermann, A. , and Buchholz, J. M. (2017b). “ The effect of nearby maskers on speech intelligibility in reverberant, multi-talker environments,” J. Acoust. Soc. Am. 141(3), 2214–2223. 10.1121/1.4979000 28372143

[56] Wiggins, I. M. , and Seeber, B. U. (2012). “ Effects of dynamic-range compression on the spatial attributes of sounds in normal-hearing listeners,” Ear Hear. 33(3), 399–410. 10.1097/AUD.0b013e31823d78fd 22246139

